# Rating of the Effectiveness of 26 Psychiatric and Seizure Medications for Autism Spectrum Disorder: Results of a National Survey

**DOI:** 10.1089/cap.2018.0121

**Published:** 2019-03-06

**Authors:** Devon M. Coleman, James B. Adams, Amy L. Anderson, Richard E. Frye

**Affiliations:** ^1^ Arizona State University, Autism/Asperger's Research Program, Tempe, Arizona.; ^2^ Barrow Neurological Institute, Phoenix Children's Hospital, Phoenix, Arizona.

**Keywords:** autism spectrum disorders, medications, psychiatric medications, seizure medications, online survey

## Abstract

***Objective:*** The objective of this study was to provide an evaluation of the benefits and adverse effects (AEs) of psychiatric and seizure medications commonly used for individuals with autism spectrum disorder (ASD).

***Methods:*** As part of the National Survey on Treatment Effectiveness for Autism, we report ratings of 26 psychiatric and seizure medications by 505 participants. Each medication was rated with a standardized scale for overall benefits, overall AEs, and specific symptoms affected. The frequency of use and net perceived benefit (overall benefit minus overall AE) are reported.

***Results:*** Most medications were rated as having a slightly greater benefit than AE. Six medications (lamotrigine, oxcarbazepine, clonidine, guanfacine, buspirone, and sertraline) had benefit ratings that were more than twice their adverse rating. Conversely, some medications had slightly negative net benefit ratings (worse AEs than benefits on average), including Adderall, Paroxetine, Quetiapine, Olanzapine, and Topiramate. However, there were wide variations in individual ratings of benefit and AEs, suggesting that clinical response to medications was highly variable, so these scores simply represent averages. A ranking of the top medications (those with the highest net perceived benefit) for each of 18 different symptoms is provided, which may provide some clinical guidance as to which medications may be most worth considering for a given symptom. A comparison of the survey results with the results of clinical trials shows generally good agreement in terms of medication benefits with some differences; in some cases the differences are because the clinical trials did not assess all of the symptoms assessed by this survey.

***Conclusions:*** It is hoped that physicians and their patients will find the survey results useful in selecting the most promising medications for a given symptom, and also for monitoring for likely benefits and AEs, especially for medications for which few or no studies have been carried out in ASD populations.

## Introduction

Autism Spectrum Disorder (ASD) is a developmental disorder that primarily involves deficits in communication and social skills and restricted/repetitive behaviors. In addition, individuals with ASD are likely to have at least one other psychiatric diagnosis (Abdallah et al. 2011; Houghton et al. 2017). Due to ASD and associated psychiatric symptoms, psychiatric medications are very commonly prescribed to children and adults with ASD. For example, a study by Houghton et al. published in 2017 found that about two-third of their ASD cohort (*n* = 93,639) had taken at least one psychiatric medication, with over one-third of the cohort having taken two or more psychiatric medications.

While psychiatric medication use is very common in ASD populations, the only ASD-related medications that are approved by the Food and Drug Administration (FDA) are risperidone and aripiprazole, which are approved for irritability associated with ASD (LeClerc and Easley 2015). There is no FDA-approved medication for treating the core symptoms of ASD (Frye and Rossignol 2016).

There have been many valuable studies on psychiatric medications for treating patients with ASD. The best studied are the atypical antipsychotic medications. Although these medications can be effective for behavior, their long-term adverse effects (AEs) are concerning. For example, the detrimental effects on lipid and glucose metabolism and body weight can develop quickly within 12 weeks (Correll et al. 2009), and long-term studies have found an increase in the risk of cardiovascular disease and type 2 diabetes (Bobo et al. 2013). There are also some concerns regarding AEs with other commonly used psychotropic medications. For example, although stimulant medications can be helpful for hyperactivity in children with ASD, the relatively high incidence of AEs alters the risk–benefit ratio of these medications (Research Units on Pediatric Psychopharmacology Autism Network 2005).

Interestingly, a recent Cochrane review has suggested that selective serotonin reuptake inhibitors (SSRIs) have no evidence for efficacy, despite promising evidence in early studies, and may actually do more harm than good (Williams et al. 2013). Thus, there is good evidence that these medications need to be studied further, particularly with respect to their effectiveness in the real-world setting, including both benefits and AEs.

One way to obtain effectiveness data on medications in the real-world setting is through questionnaire survey methods. Several such studies have been conducted in children with ASD using simple Likert scales. Goin-Kochel et al. (2009) conducted an online survey of 479 parents/caregivers of children with ASD in 2008, examining the effectiveness of 70 medications and 16 behavioral and educational therapies. Owen-Smith et al. (2015) performed a survey study on 42 complementary and alternative medicines used by individuals with ASD. Frye et al. (2011) surveyed the effectiveness of treatment for seizures in children with ASD.

However, by far, the largest medication survey, the Parent Ratings of Behavioral Effects of Biomedical Interventions Survey, was conducted by the Autism Research Institute (ARI) and published in 2008. In this survey 27,000 parents of children with ASD rated the effectiveness of 84 different drugs, supplements, and diets on a six-point scale from “made worse” to “made better.” Seven of the drugs used for seizures demonstrated their effect both on behavior and on seizures (Autism Research Institute 2009).

While all of these surveys successfully collected effectiveness data for medications used in ASD populations, they mostly used an overall rating, rather than a separate rating for benefits and a separate rating for AEs. Also, most surveys did not obtain information on specific symptoms. To improve upon previous research, we designed a survey that separated the scales for overall benefits and overall AEs, while also obtaining information about specific benefits and AEs. Analysis of the data allows an estimate of which medications are perceived to be the most beneficial for a given symptom, with 18 beneficial symptoms being evaluated.

The data reported here are from the National Survey on Treatment Effectiveness for Autism, which evaluated the effects of all medications, supplements, diets, therapies, and educational interventions, but this article is only reporting on the Psychiatric and Seizure medications data.

## Methods

### Survey content and development

The National Survey on Treatment Effectiveness for Autism (from now on referred to as “the survey”) was created over several months by the research team. A draft version of the survey was created, and then reviewed by several autism families and by several autism experts, including physicians, nutritionists, board-certified behavior analysts, and educational experts. An initial version was then developed using Survey Gizmo, and tested with several autism families; based on that feedback, the survey was significantly modified to reduce the time required to complete it. A revised version was then shared with 10 autism families to obtain feedback on usability and appropriateness of symptoms and terminology used. Based on their feedback, a few terms were changed, but overall the survey was found to be easy to use and understand.

**FIG. 1.**
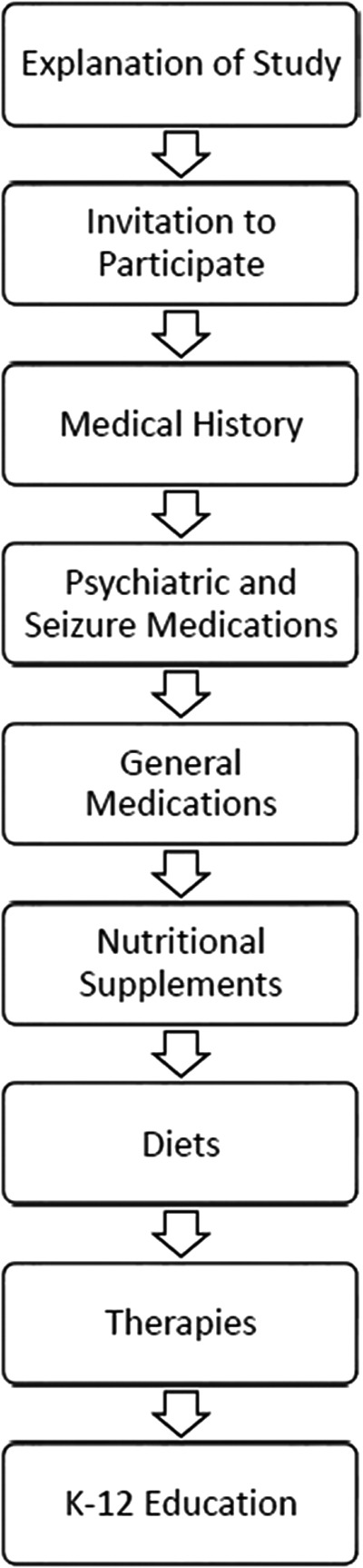
Flow chart of survey.

The survey consists of seven parts: general medical history, psychiatric and seizure medications, general medications, nutritional supplements, diets, therapies, and K-12 education (Fig. 1). This article only reports the results on the medical history section and the psychiatric and seizure medications, with the results of the other sections to be published in additional papers.

The medical history section gathered information on the person with ASD (from now on called “the participant”), including their age, gender, diagnosis, developmental history, and severity of autism-related symptoms.

For the psychiatric and seizure medication section, the survey first asked what psychiatric or seizure medications the participant had taken (from a list of 63 common medications). For any medication selected, the survey then asked for the overall benefit of the medication (no benefit = 0, slight benefit = 1, moderate benefit = 2, good benefit = 3, or great benefit = 4), the primary symptoms benefited, the overall AE of the medication (no AEs = 0, mild AEs = 1, moderate AEs = 2, or severe AEs = 3), and the specific symptoms that were adversely effected. Table 1 shows the symptom list from which participants could select (they could select one or more for each medication). Finally, at the end of the survey participants were asked, “Overall, what benefit do you think psychiatric medication had on the participant” and “Overall, what benefit do you think seizure medications had on the participant.”

**Table 1.** All Symptom Options

*Benefited symptom options*	*Adverse symptom options*
General benefit	General worsening
Aggression/agitation	Aggression/agitation
Anxiety	Anxiety
Attention	Bedwetting/bladder control
Cognition (ability to think)	Behavior problems
Constipation	Cognition (ability to think)
Depression	Depression
Diarrhea	Dizziness/unsteadiness
Eczema/skin problem	Dry mouth
Health (fewer illnesses and/or less severe illnesses)	Fatigue/drowsiness
	Gastrointestinal problems
Hyperactivity	Headache/migraine
Irritability	Hyperactivity
Language/communication	Irritability
Lethargy (easily tired)	Liver/kidney problem
OCD	Loss of appetite
Reflux/vomiting	Nausea
Seizures	Rash
Self-injury	Seizures
Sensory sensitivity	Self-injury
Sleep (falling asleep)	Sleep problems
Sleep (staying asleep)	Stimming/perseveration
Social interaction and understanding	Tics/abnormal movements
Stimming/perseveration	Weight gain
Tics/abnormal movements	Weight loss

OCD, obsessive-compulsive disorder.

### Survey distribution

After approval by the Institutional Review Board (IRB) of Arizona State University, the survey was advertised to autism families across the country. The study was advertised with the help of 60 autism organizations, including the Autism Society, 30 chapters of the Autism Society, Autism Speaks, the Autism Research Institute, and many other groups (see list in “Acknowledgments”), as well as our own email list for autism families interested in our research. The survey was also advertised across the country with Facebook ads; this was one of the most effective advertising mechanisms. All ads were IRB approved, and were linked to a website that posted the online survey and an invitation to participate. Completing the online survey was accepted as informed consent.

Over 6 months, 1079 people filled out the survey. Of the 882 who filled out the psychiatric and seizure medication section, 378 responses indicated that they had not taken psychiatric or seizure medications, leaving 505 participants who rated psychiatric and seizure medications.

### Data analysis

Results were collected through Survey Gizmo and then exported into Microsoft Excel. Data are shown only for medications for which there were ≥20 responses (used by >5% of respondents), so that only 26 medications of 63 are reported here. Data were analyzed in two different ways: by medication and by symptom. For the medication analysis, we report the overall benefit and overall adverse score for each medication, which were averages of the ratings by each participant. We calculate the net benefit defined as overall benefit minus overall adverse. Also, for each symptom, there was a calculation of the percentage of people who believe that the symptom was affected out of the total number of people who rated that medication.

For the symptom analysis, a medication “effectiveness score” was calculated for each medication's effect on a particular symptom. The score was calculated by multiplying the medication's net benefit rating by the percentage of responses that said that the medication benefited the symptom in question. For example, if the average benefit rating of a medication was 1.3, and if 50% of people using that medication reported a primary benefit for a particular symptom like sleep, then the sleep rating would be 1.3 × 50% = 0.65. This allows for approximate comparisons of medications with one another.

## Results

### Demographics and medical history

The characteristics of the participants and their medical history are listed in Table 2. Most of the surveys were completed by a primary caregiver (87%). Respondents were spread fairly evenly across the United States, with the most respondents from Arizona, California, and New York. Half of the surveys were for children (54%), but many were for teens (21%) and adults (25%). Seventy-seven percent of the participants were male, and 23% were female, similar to many other studies (Fombonne 2003). Autism was the most common diagnosis (42%), followed by ASD (21%) and Asperger's (16%). Respondents who said they did not have an official diagnosis were removed from the analysis and not included in this article. Thirty-four percent of participants had early onset of symptoms, but 56% had normal development followed by a plateau or regression. Regression occurred at an average age of 19.3 months, and usually affected all three core areas of ASD (communication, social interaction, and behavior), and sometimes motor skills. The perceptions of possible causes of the regression are listed in Table 2.

**Table 2.** Participant Medical Histories

Survey completed by (*n* = 886)	
Primary caregiver of an individual with autism	87%
Adults with autism and their mother/father/childhood guardian	3%
Adult with high-functioning autism >18 years old who doesn't have a guardianship	5%
Grandparent of an individual with autism	4%
Location (by region)	
Midwest	20%
Northeast	22%
South	31%
West	27%
Age of participants (years)	
Child (<13)	54%
Teenager (13–18)	21%
Young adult (19–30)	17%
Adult (>30)	8%
Gender of participants	
Male	77%
Female	23%
Current medical diagnosis	
Autism spectrum disorder (this is less severe than a diagnosis of autism)	21%
Asperger's syndrome	16%
Autism	42%
High-functioning autism	12%
No current diagnosis, but he/she was on the autism spectrum previously	2%
Pervasive developmental disorder-not otherwise specified (PDD-NOS)	7%
Developmental history	
Normal development, followed by a plateau in development that lasted for several months or longer	22%
Normal development, followed by a major regression and a plateau lasting several months or longer	13%
Normal development, followed by major regression	21%
Abnormal development from early infancy, with no major regression or plateau in development	34%
Other	10%
Regression information	
Age of regression (in months)	
Average	19
First quartile	12
Third quartile	24
Skills primarily affected by regression	
Language	84%
Social interactions	82%
Behavior	81%
Motor skills	46%
Perceived cause of regression (more than one response was allowed)	
High fever	11%
Illness	8%
Seizure	6%
Vaccination	51%
Unknown	45%
Other	15%
Number of regressions (if they had a regression)	
1	48%
2	18%
3	11%
4–5	6%
≥6	6%
Perceived triggers for the regressions (if they said they had more than one regression)	
High fever	5%
Illness	8%
Seizure	6%
Vaccination	35%
Unknown	35%
Other	8%
Genetic conditions	
No genetic testing done	60.0%
Genetic testing normal	29.4%
Angelman's syndrome	0.2%
Down's syndrome	0.5%
Fragile X	1.5%
PTEN	0.1%
Prader-Willi syndrome	0.0%
Rett's syndrome	0.0%
Smith-Lemli-Opits syndrome	0.0%
Tuberous sclerosis	0.0%
Other microarray abnormality	1.8%
Other genetic disorder	6.5%
Metabolic disorders	
No metabolic abnormalities	47.1%
No metabolic testing done	44.8%
Mitochondrial disease (due to genetic abnormality)	1.3%
Mitochondrial dysfunction (not due to known genetic cause)	2.7%
Cerebral folate deficiency	1.7%
Carnitine abnormalities	1.4%
Urea cycle defect	0.2%
Purine metabolic defect	0.4%
Sulfation defect	0.8%
MTHFR abnormality	5.2%
Other	4.4%
Rounds of antibiotic usage within first 3 years (10 days = 1 round)	
Average	7.2
Median	3.0
0 Rounds	14%
1 Round	18%
2 Rounds	12%
3 Rounds	16%
4 Rounds	6%
5 Rounds	6%
6–7 Rounds	9%
8–10 Rounds	8%
11–15 Rounds	3%
16–20 Rounds	3%
21+ Rounds	7%
Severity of autism-related symptoms at age 3	
No autistic symptoms	4%
Nearly normal, with only very mild symptoms	18%
Mild autism	24%
Moderate autism	37%
Severe autism	17%
Severity of autism-related symptoms currently	
No autistic symptoms	1%
Nearly normal, with only very mild symptoms	16%
Mild autism	30%
Moderate autism	39%
Severe autism	14%

PTEN, phosphatase and tensin homolg; MTHFR, methylenetetrahydrofolate reductase.

Regarding genetic disorders, ∼10% reported a genetic disorder, 29% reported no genetic abnormalities, and in 60% of participants no genetic testing was done—so the percentage with genetic disorders is likely higher than that reported due to lack of testing. Approximately 7% reported a metabolic disorder, but in 45% of participants no metabolic testing was done, so the percentage with metabolic disorders is likely higher than that reported.

Antibiotics were used in the first 3 years of life by 84% of participants, with an average of seven rounds (median of three) across all participants (one round = 10 days), which is somewhat higher than that reported in several other studies for individuals with ASD, and much higher than that reported for typically developing individuals in those studies (Konstantareas and Homatidis 1987; Adams et al. 2006, 2007, 2008; Niehus and Lord 2006). Although the question specified that the answer should be in rounds with one round equaling 10 days, it is possible that a few people responded with the number of days, therefore skewing our numbers to be slightly higher than those of other studies.

Table 3 lists autism severity at age 3 and the autism severity currently (age ≥10), as estimated by the respondent. For those participants with no symptoms or nearly normal, at age 3, their current symptoms were mostly nearly normal or mild autism. For those participants with mild autism at age 3, their current symptoms were also mostly mild autism, with some slightly improving or worsening. For the participants with moderate autism at age 3, most still had moderate autism, with some slightly improving and a few worsening. For those with severe autism at age 3, most still had severe autism, but some improved. So, initial autism severity at age 3 was a strong predictor of autism severity at later ages.

**Table 3.** Autism Spectrum Disorder Symptom Severity at Age 3 Versus Now

		*Current*				
		*No autistic symptoms (%)*	*Nearly normal, with only very mild symptoms (%)*	*Mild autism (%)*	*Moderate autism (%)*	*Severe autism (%)*
Age 3	No autistic symptoms (39)	3	41	41	15	0
	Nearly normal, with only very mild symptoms (123)	2	40	35	20	4
	Mild autism (126)	0	16	47	31	6
	Moderate autism (190)	3	9	21	58	8
	Severe autism (98)	2	5	14	23	55

### Psychiatric and seizure medications

The psychiatric and seizure medications fall into five general categories: stimulants (four medications), SSRIs (five medications), antipsychotics (four medications), seizure (nine medications), and other (four medications) (Table 4).

**Table 4.** Psychiatric and Seizure Medications

*Treatment (*n*)*	*Overall benefit score*	*Top benefited symptoms*	*Overall adverse score*	*Top adverse symptoms*	*Category*
Clonidine/Catapres/Kapvay (99)	1.9	Falling asleep (49%), staying asleep (32%), anxiety (17%)	0.7	Fatigue/drowsiness (12%), aggression/agitation (9%), behavior problems (9%), anxiety (7%), dizziness/unsteadiness (7%), sleep problems (7%), gastrointestinal problems (6%), irritability (6%), general worsening (5%)	Other
Lamotrigine/Lamictal (34)	2.1	Seizures (32%), aggression/agitation (24%), anxiety (12%), irritability (12%)	0.9	Sleep problems (9%), weight gain (9%), aggression/agitation (6%), cognition (6%), depression (6%), dizziness/unsteadiness (6%), fatigue/drowsiness (6%), gastrointestinal problems (6%), headache/migraine (6%)	Antiseizure
Guanfacine/Intuniv/Tenex (96)	1.7	Attention (39%), hyperactivity (25%), aggression/agitation (20%), cognition (20%)	0.7	Fatigue/drowsiness (16%), irritability (9%), aggression/agitation (8%)	Other
Oxcarbazepine/Oxtellar/Trileptal (29)	1.9	Aggression/agitation (31%), seizures (28%), anxiety (24%)	0.8	Anxiety (17%), irritability (14%), behavior problems (10%), cognition (10%)	Antiseizure
Sertraline/Zoloft (101)	1.6	Anxiety (56%), depression (24%), aggression/agitation (14%), irritability (14%)	0.6	General worsening (9%), depression (8%), weight gain (7%)	SSRI
Diazepam/Valium (28)	1.6	Anxiety (36%), seizures (14%), aggression/agitation (11%)	0.8	Fatigue/drowsiness (14%), general worsening (11%), aggression/agitation (7%), anxiety (7%), depression (7%), dizziness/unsteadiness (7%), sleep problems (7%)	Antiseizure
Buspirone/Buspar/Vanspar (42)	1.3	Anxiety (45%), aggression/agitation (14%), irritability (12%)	0.5	Anxiety (12%), fatigue/drowsiness (10%), aggression/agitation (7%)	Other
Citalopram/CeleXA (38)	1.5	Anxiety (45%), depression (29%), irritability (11%)	0.7	Aggression/agitation (16%), anxiety (13%), behavior problems (13%)	SSRI
Fluoxetine/Prozac/Sarafem/Rapidflux (102)	1.5	Anxiety (44%), depression (23%), aggression/agitation (18%)	0.8	Aggression/agitation (10%), cognition (10%), irritability (10%)	SSRI
Aripiprazole/Abilify (99)	1.6	Aggression/agitation (47%), irritability (25%), anxiety (22%)	0.9	Weight gain (23%), aggression/agitation (16%), tics/abnormal movements (9%)	Antipsychotic
Escitalopram/Lexapro (40)	1.3	Anxiety (35%), depression (33%), general benefit (13%), aggression/agitation (13%), irritability (13%)	0.8	Aggression/agitation (10%), anxiety (10%), cognition (8%), depression (8%), dry mouth (8%), irritability (8%), weight gain (8%)	SSRI
Levetiracetam/Keppra (25)	1.6	Seizures (68%), aggression/agitation (4%), anxiety (4%), cognition (4%), language/communication (4%), social interaction and understanding (4%)	1.1	Aggression/agitation (28%), behavior problems (28%), anxiety (24%)	Antiseizure
Amphetamine (21)	1.3	Attention (43%), hyperactivity (38%), anxiety (14%), cognition (14%), irritability (14%)	0.9	Loss of appetite (19%), sleep problems (19%), anxiety (14%), behavior problems (14%), tics/abnormal movements (14%)	Stimulant
Dexmethylphenidate/Focalin (53)	1.5	Attention (43%), hyperactivity (26%), cognition (23%)	1.1	Aggression/agitation (21%), irritability (21%), loss of appetite (21%)	Stimulant
Divalproex Sodium/Depakote (56)	1.3	Seizures (20%), aggression/agitation (16%), irritability (16%)	0.9	Weight gain (13%), depression (11%), fatigue/drowsiness (11%)	Antiseizure
Clonazepam/Klonopin (68)	1.3	Anxiety (25%), falling asleep (19%), staying asleep (10%)	1.0	Fatigue/drowsiness (18%), aggression/agitation (15%), anxiety (10%), behavior problems (10%)	Antiseizure
Atomoxetine/Strattera (67)	1.1	Attention (28%), anxiety (21%), aggression/agitation (15%), hyperactivity (15%)	0.8	Aggression/agitation (19%), behavior problems (15%), irritability (15%)	Other
Carbamazepine/Tegretol/Carbatrol Epitol (28)	1.1	Seizures (29%), anxiety (14%), irritability (14%)	0.8	Fatigue/drowsiness (14%), aggression/agitation (11%), dizziness/unsteadiness (11%), tics/abnormal movements (11%)	Antiseizure
Risperidone/Risperdal (170)	1.6	Aggression/agitation (40%), irritability (24%), anxiety (21%)	1.4	Weight gain (35%), aggression/agitation (14%), behavior problems (11%)	Antipsychotic
Gabapentin/Neurontin/Gralise/Horizant (25)	1.0	Anxiety (16%), general benefit (8%), irritability (8%)	0.9	General worsening (16%), anxiety (16%), cognition (12%), depression (12%), dizziness/unsteadiness (12%), seizures (12%)	Antiseizure
Methylphenidate/Ritalin/Medate/Concerta (117)	1.4	Attention (48%), hyperactivity (39%), cognition (23%)	1.2	Aggression/agitation (26%), anxiety (23%), loss of appetite (22%), irritability (20%)	Stimulant
Quetiapine/Seroquel (47)	1.0	Aggression/agitation (28%), falling asleep (23%), staying asleep (17%)	1.1	Fatigue/drowsiness (26%), weight gain (17%), cognition (15%)	Antipsychotic
Paxil/Paroxetine (20)	0.8	Anxiety (20%), attention (15%), depression (10%), social interaction, and understanding (10%)	0.8	Depression (20%), irritability (20%), aggression/agitation (15%), anxiety (15%), headache/migraine (15%), hyperactivity (15%), weight gain (15%)	SSRI
Topiramate/Qudexy/Topamax (26)	1.2	Seizures (25%), aggress/agitation (18%), depression (15%), dizziness/unsteadiness (15%), irritability (15%)	1.3	Aggression/agitation (19%), depression (15%), dizziness/unsteadiness (15%), irritability (15%)	Antiseizure
Adderall (152)	1.0	Attention (39%), hyperactivity (23%), cognition (18%)	1.3	Aggression/agitation (36%), anxiety (21%), behavior problems (20%), irritability (20%)	Stimulant
Olanzapine/Zyprexa (22)	1.0	Aggression/agitation (32%), anxiety (32%), hyperactivity (18%)	1.5	Weight gain (36%), aggression/agitation (18%), cognition (18%), irritability (18%)	Antipsychotic

The medications are listed in order of net benefit (highest to lowest).

*n* refers to the number of participants who reported using the medication. For top benefited symptoms and top adverse symptoms, the percentage is the fraction of respondents who selected the symptom as a primary benefit/adverse effect.

SSRI, selective serotonin reuptake inhibitor.

**FIG. 2.**
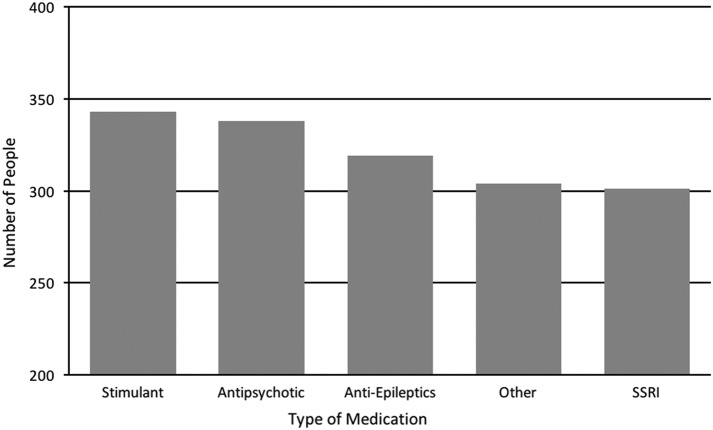
Number of people who used one or more medications in each category, out of a total of 505 people who used medications. SSRI, selective serotonin reuptake inhibitor.

The most commonly used psychiatric medications were stimulants, closely followed by antipsychotics, seizure medications, and SSRIs (Fig. 2). The number of psychiatric medications that participants had taken increased with age, with children (0–12 years) having taken an average of 1.4, teenagers (13–18 years) having taken an average of 3.1, and adults (19+ years) having taken an average of 4 (Table 5). In all the discussions shown below about other studies, the studies involved individuals with ASD unless otherwise stated.

**Table 5.** Number of Psychiatric Medications Used

*Number of psychiatric medications*	N	*%*
Child (0–12 years)		
0	270	59
1	71	15
2	42	9
3	18	4
4	13	3
5	11	2
6	6	1
7	6	1
8	9	2
9	2	0
10+	11	2
Average	1.4	
Teen (13–18 years)		
0	46	25
1	36	20
2	17	9
3	24	13
4	18	10
5	9	5
6	10	5
7	8	4
8	1	1
9	2	1
10+	13	7
Average	3.1	
Adult (19+ years)		
0	40	18
1	34	16
2	30	14
3	25	11
4	17	8
5	20	9
6	13	6
7	7	3
8	7	3
9	7	3
10+	18	8
Average	4.0	

**FIG. 3.**
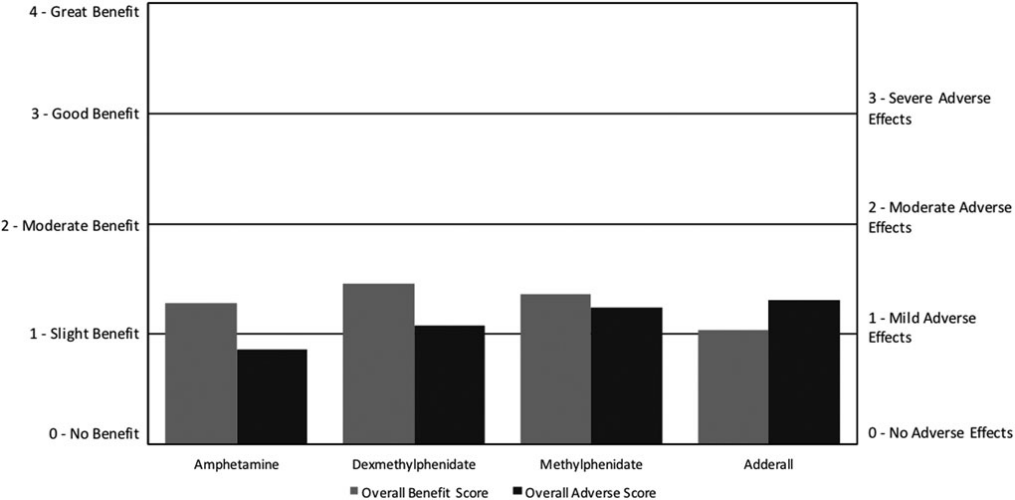
Overall benefit score and adverse score for stimulant medications. For overall benefit, the scoring was no benefit (0), slight benefit (1), moderate benefit (2), good benefit (3), or great benefit (4). For overall AEs, the rating was no AEs (0), mild AEs (1), moderate AEs (2), or severe AE (3). AE, adverse effect.

For stimulants, the overall benefit scores ranged from 1.0 to 1.5, while the overall adverse scores had a similar range from 0.9 to 1.3. The most frequently used stimulant was amphetamine/dextroamphetamine (Adderall), followed closely by methylphenidate, with dexmethylphenidate and amphetamine used much less frequently. Stimulants had only slight benefit on average, with overall benefit scores ranging from 1.0 to 1.5 (on a scale of 0–4, with slightly better = 1, better = 2), and overall adverse scores ranging from 0.9 to 1.3—only slightly lower than the overall benefit scores. Amphetamine had the highest net benefit (benefit minus adverse), followed by dexmethylphenidate, methylphenidate, and amphetamine/dextroamphetamine (Adderall) (Fig. 3; Table 4). In comparison, the ARI survey found that amphetamine/dextroamphetamine (Adderall), methylphenidate, and amphetamine all had negative net benefits (Autism Research Institute 2009).

While amphetamine/dextroamphetamine (Adderall) was the most commonly used stimulant, it had the lowest net benefit with the overall adverse score being higher than the overall benefit. This survey found that all four medications were reported to help with attention, hyperactivity, and cognition, similar to a meta-analysis of four randomized, double-blind, placebo-controlled trials (RDBPCTs) of methylphenidate, which reported significant improvements in hyperactivity (there were no published studies for the other medications with ASD populations). This survey found similar AEs among the four medications, including primarily loss of appetite, aggression/agitation, irritability, behavior problems, and sleep problems. This is somewhat similar to the meta-analysis of methylphenidate, which found that it primarily caused decreased appetite and insomnia (Reichow et al. 2013).

**FIG. 4.**
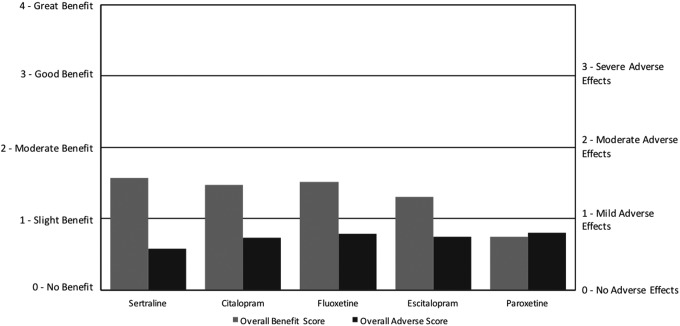
Overall benefit score and adverse score for selective serotonin reuptake inhibitor medications.

For SSRIs, the overall benefit scores ranged from 0.8 to 1.6, while the overall adverse scores ranged from 0.6 to 0.8. Fluoxetine and sertraline were the most commonly used, with escitalopram, citalopram, and paroxetine used much less frequently. Sertraline had the highest net benefit followed by citalopram, fluoxetine, escitalopram, and finally paroxetine, which had zero net benefit (Fig. 4; Table 4). For the ARI survey, it was found that fluoxetine and paroxetine had positive, although small, net benefits, while sertraline had a negative net benefit (Autism Research Institute 2009). All five medications primarily benefited anxiety, with some benefit for depression, aggression/agitation, and irritability. The most common AEs were aggression/agitation, anxiety, irritability, depression, weight gain, and cognition.

Research studies have found somewhat similar, but varying, benefits and AEs of SSRIs on ASD populations. For sertraline, a systematic review of three prospective, open-label studies indicated that it significantly improved aggression, repetitive behaviors, and anxiety, but often caused weight gain and anxiety/agitation (Kolevzon et al., 2006). For citalopram, a large randomized, placebo-controlled trial concluded that there was a significant improvement in irritability, but that serious adverse events such as hyperactivity and stereotypy were more frequent compared with placebo (King et al. 2009). For fluoxetine, three RDBPCTs found that it significantly improved anxiety, Clinical Global Impressions scores, and some obsessive-compulsive disorder (OCD) behaviors, while causing “relatively few side effects,” mainly insomnia (Williams et al. 2013). For Escitalopram, an open-label pharmacogenetics study found that it significantly improved irritability, but adverse events were not documented (Bishop et al. 2015). There were no studies for Paroxetine in ASD populations.

**FIG. 5.**
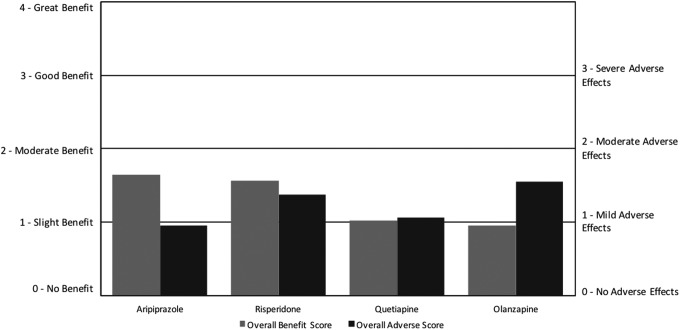
Overall benefit score and adverse score for antipsychotic medications.

For antipsychotics, the overall benefit scores ranged from 1.0 to 1.6, with a similar range of overall adverse scores from 0.9 to 1.5. Risperidone and aripiprazole were the most commonly used, with quetiapine and olanzapine used much less frequently. Aripiprazole had the highest net benefit followed by risperidone, quetiapine, and finally olanzapine, with the latter two medications having negative net benefit scores (Fig. 5; Table 4). For the ARI survey, it was found that risperidone had a large net benefit; however, it did not include data on the other three medications (Autism Research Institute 2009).

All four medications primarily benefited aggression/agitation, irritability, and anxiety. All four primarily adversely affected weight gain, aggression/agitation, cognition, fatigue/drowsiness, irritability, behavior problems, and tics/abnormal movements. These results are similar to several RDBPCTs (Sochocky et al., 2013; Fung et al. 2016) and open-label studies (Sochocky et al., 2013; Masi et al. 2015) on all four antipsychotics, which found the benefits to primarily be reductions of aggression/agitation and irritability, and AEs to be weight gain and sedation.

**FIG. 6.**
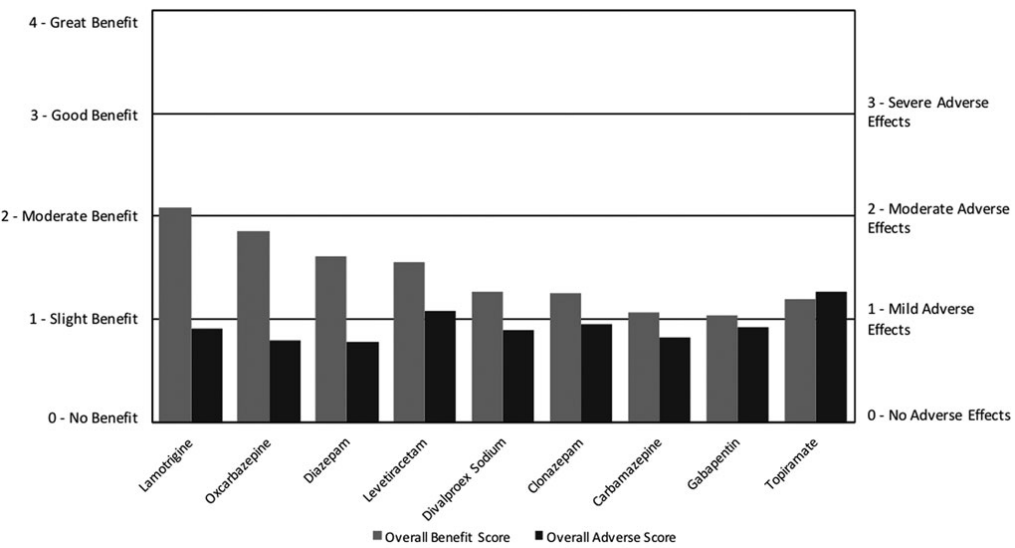
Overall benefit score and adverse score for seizure medications.

For seizure medications, the overall benefit scores ranged from 1.0 to 2.1 with overall adverse scores ranging from 0.8 to 1.3. Clonazepam and valproate were the most commonly used, with lamotrigine, oxcarbazepine, carbamazepine, diazepam, topiramate, levetiracetam, and gabapentin used much less frequently. Lamotrigine and oxcarbazepine had the highest net benefit followed by diazepam, levetiracetam, valproate, clonazepam, carbamazepine, gabapentin, and finally topiramate, which had a slightly negative net benefit (Fig. 6; Table 4). The ARI survey found that divalproex sodium and carbamazepine both had high net benefit for seizures and behavioral symptoms, whereas clonazepam and diazepam both had negative net benefit. The other medications listed above were not included in the ARI survey (Autism Research Institute 2009).

Carbamazepine, diazepam, divalproex sodium, lamotrigine, levetiracetam, and oxcarbazepine all primarily improved seizures, anxiety, and aggression. Clonazepam primarily improved anxiety and sleep, gabapentin primarily improved anxiety, and topiramate primarily improved aggression/agitation and cognition, but none of these three substantially improved seizures. A variety of AEs were reported, with the most common among all seizure medications being anxiety, aggression/agitation, and fatigue/drowsiness. Interestingly, a few participants reported that gabapentin caused seizures.

Research studies found varied and inconsistent results on benefits and AEs of the different seizure medications on ASD populations. For levetiracetam, a systematic review of one RDBPCT and one open-label study indicated that it improved seizures, hyperactivity, inattention, and aggression but often caused aggression or behavioral changes. However, the results were inconsistent across studies (Frye et al. 2013). For topiramate, a systematic review of one RDBPCT and four open-label studies indicated that it significantly improved irritability, hyperactivity, stereotypy, and, to a lesser degree, anxiety and depression, but often caused decreased appetite, agitation, hyperactivity, and cognitive difficulties (Doyle and Mcdougle 2012). A review of seizure medications for ASD populations found that valproate was recommended for seizures, and well recommended for behavioral symptoms; carbamazepine, clonazepam, and lamotrigine were recommended for seizures, but not for behavioral symptoms; and that oxcarbazepine and gabapentin were minimally recommended for seizures, but had no studies showing a benefit for behavioral symptoms (Frye et al. 2013). There were no published studies on the benefit of diazepam on ASD symptoms.

**FIG. 7.**
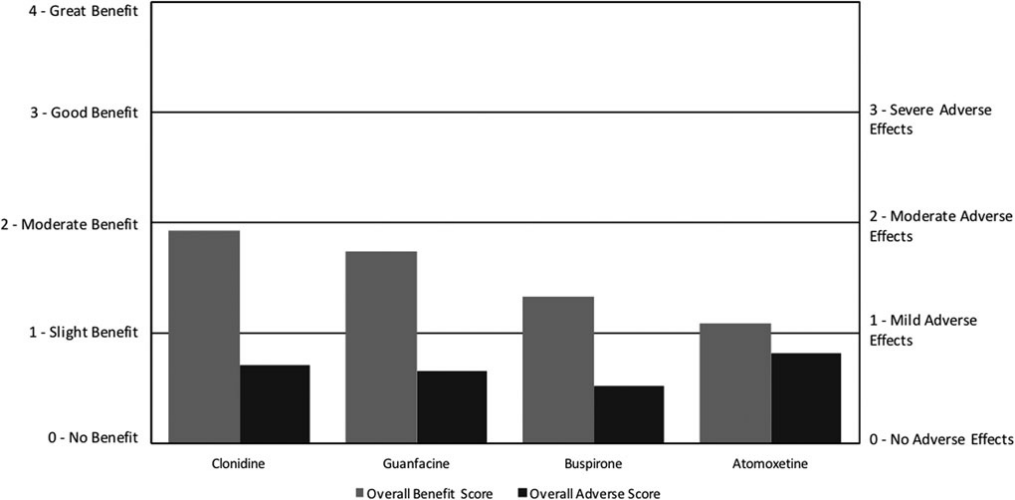
Overall benefit score and adverse score for other psychiatric medications.

There were a few medications included in the survey that did not belong to the previously listed categories, so we included them in the “Other” category. Among these medications, clonidine and guanfacine were the most commonly used, with atomoxetine and then buspirone being used less. For the Other psychiatric medications, clonidine had the highest net benefit, followed by guanfacine, buspirone, and then atomoxetine (Fig. 7; Table 4). For the ARI survey, clonidine had a high net benefit, while buspirone had a negative net benefit. The other two medications were not included in the ARI survey (Autism Research Institute 2009).

Clonidine primarily benefited falling asleep, staying asleep, and anxiety, but had a variety of AEs, including fatigue/drowsiness, aggression/agitation, and behavior problems. In contrast, a RDBPCT found that clonidine improved irritability, hyperactivity, and stereotypy, and caused drowsiness and fatigue (no report of effect on sleep or anxiety) (Jaselskis et al. 1992). An open-label, retrospective study did find that clonidine helped in sleep initiation and night walking, but did not evaluate possible improvements in anxiety (Ming et al. 2008).

Guanfacine primarily improved attention, hyperactivity, aggression/agitation, and cognition, and adversely affected fatigue/drowsiness and irritability. These results are similar to the results obtained from a systematic review of a RDBPCT crossover trial and an open-label study, which indicated that guanfacine significantly improved hyperactivity and inattention but caused adverse events such as irritability and drowsiness (Wink et al. 2010).

Buspirone primarily benefited anxiety, aggression/agitation, and hyperactivity, while adversely affecting anxiety, fatigue/drowsiness, and aggression/agitation. These results are slightly similar to a RDBPCT that indicated that buspirone significantly improved irritability, while causing drowsiness, fatigue, and increased appetite; however, the study did not look for improvements in aggression or anxiety (Ghanizadeh and Ayoobzadehshirazi 2015).

Atomoxetine primarily improved attention, anxiety, aggression/agitation, and hyperactivity while adversely affecting aggression/agitation, behavior problems, and irritability. These results are similar to those obtained from a systematic review of one RDBPCT, four open-label studies, and one retrospective study that indicated that atomoxetine significantly improved hyperactivity and inattention, but often caused irritability, gastrointestinal problems, and fatigue. None of these studies evaluated atomoxetine's effect on aggression or anxiety, which were found to be a common benefit in this survey (Ghanizadeh 2012).

A final question in the survey asked participants about their overall rating of effect of psychiatric and seizure medications (Table 6). Participants who had used these medications believed that they had a positive effect, with 76% and 86% revealing that psychiatric and seizure medications (respectively) had an overall benefit, with 15% and 11% (respectively) revealing that they had an overall negative effect.

**Table 6.** Rating of Overall Effect of Psychiatric and Seizure Medications

	*Psychiatric*	*Seizure*
Average	1.28	1.15
Responses (%)		
Much better	29	25
Somewhat better	26	24
Slightly better	21	19
No effect	9	22
Mildly worse	4	2
Somewhat worse	5	3
Much worse	6	6

**FIG. 8.**
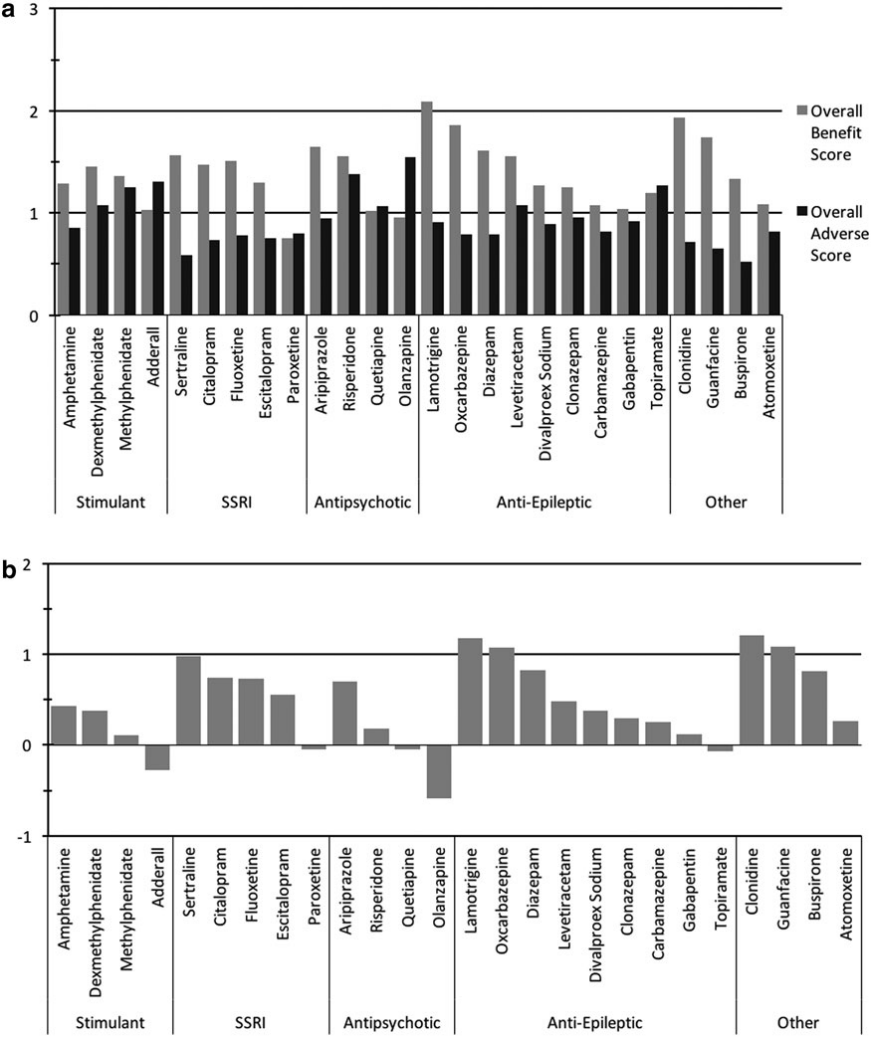
Overall benefit score and adverse score for all psychiatric and seizure medications. **(a)** Overall benefit and adverse scores. **(b)** Net benefit scores.

When considering all medications, overall benefit scores varied from 0.8 to 2.1 with an average of 1.4, and overall adverse scores varied from 0.5 to 1.5, with an average of 0.9; so, most medications achieved an overall benefit of ∼1 (slight benefit), with only a few approaching a score of 2 (moderate benefit) (Fig. 8a). Net benefit scores varied across medications in a range of 1.2 to −0.6, with an average net benefit score of 0.5 (Fig. 8b).

**FIG. 9.**
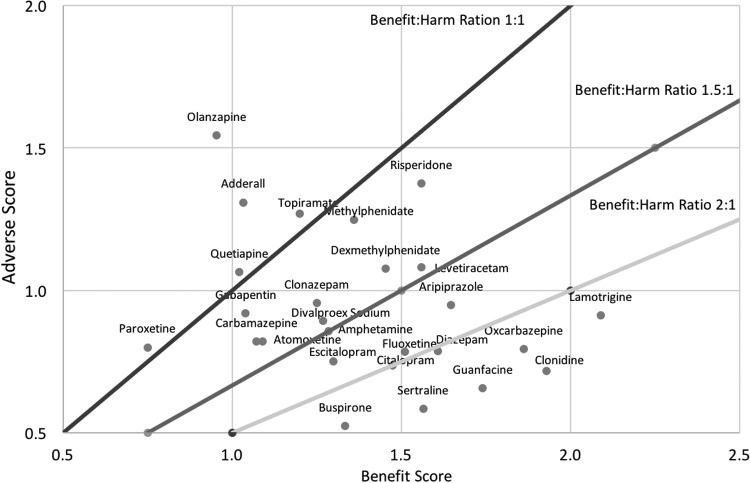
Benefit:harm ratio of all psychiatric medications. Plot of overall AE versus overall benefit for all medications. There are three lines indicating the ratio of overall benefit to overall AE for ratios of 1:1, 1.5:1, and 2:1. Medications on the *lower right* have the highest ratio of overall benefit to overall AE.

The benefit-to-harm ratio of the psychiatric medications significantly varied. Some medications such as lamotrigine, clonidine, guanfacine, sertraline, and buspirone had overall benefit scores that were more than twice the overall adverse rating, while others such as olanzapine, amphetamine/dextroamphetamine (Adderall), paroxetine, quetiapine, and topiramate had negative net benefit score (Fig. 9).

### Top medications for different symptoms

Table 7 shows the top-rated medications for 18 different symptoms. For some symptoms, psychiatric and/or seizure medications are moderately effective (net benefit scores >0.25), including symptoms of aggression/agitation, anxiety, attention, falling asleep, hyperactivity, seizures, and staying asleep. Other symptoms were slightly affected (net benefit scores between 0.10 and 0.25) by psychiatric and/or seizure medications, such as cognition, depression, irritability, OCD, sensory sensitivity, social interaction and understanding, and tics/abnormal movements. Finally, several symptoms were not significantly affected by psychiatric and/or seizure medications (net benefit scores <0.10), including general benefit, self-injury, lethargy, stimming/perseveration/desire for sameness, and language/communication (Table 7).

**Table 7.** Top Medications for Symptoms

*Symptoms*	*Medication (benefit rating)*
Aggression/agitation	Oxcarbazepine (0.33), Lamotrigine (0.28), Guanfacine (0.21), Aripiprazole (0.16), Clonidine (0.16), Sertraline (0.14), Fluoxetine (0.13), Buspirone (0.12)
Anxiety	Sertraline (0.55), Buspirone (0.38), Citalopram (0.33), Fluoxetine (0.32), Diazepam (0.29), Oxcarbazepine (0.26), Clonidine (0.21), Escitalopram (0.19), Guanfacine (0.18), Lamotrigine (0.14)
Attention	Guanfacine (0.42), Amphetamine (0.20), Dexmethylphenidate (0.16), Clonidine (0.11)
Cognition (ability to think)	Guanfacine (0.21), Dexmethylphenidate (0.09), Sertraline (0.07)
Depression	Sertraline (0.23), Citalopram (0.21), Escitalopram (0.18), Fluoxetine (0.16)
General benefit, no one particular symptom	Clonidine (0.09), Lamotrigine (0.07), Escitalopram (0.07)
Hyperactivity	Guanfacine (0.27), Clonidine (0.20), Amphetamine (0.18), Oxcarbazepine (0.11), Dexmethylphenidate (0.10), Sertraline (0.10)
Irritability	Oxcarbazepine (0.18), Lamotrigine (0.14), Sertraline (0.14), Guanfacine (0.12), Clonidine (0.12), Fluoxetine (0.10), Buspirone (0.10)
Language/communication	Sertraline (0.04), Guanfacine (0.03), Divalproex Sodium (0.02)
Lethargy (easily tired)	Diazepam (0.06), Clonidine (0.02), Buspirone (0.02)
OCD	Sertraline (0.12), Fluoxetine (0.08), Citalopram (0.08)
Seizures	Lamotrigine (0.38), Levetiracetam (0.33), Oxcarbazepine (0.29), Diazepam (0.12)
Self-injury	Lamotrigine (0.07), Buspirone (0.04), Citalopram (0.04)
Sensory sensitivity	Oxcarbazepine (0.11), Guanfacine (0.07), Amphetamine (0.05)
Sleep (falling asleep)	Clonidine (0.59), Guanfacine (0.11), Clonazepam (0.06)
Sleep (staying asleep)	Clonidine (0.39), Guanfacine (0.08), Lamotrigine (0.07)
Social interaction and understanding	Guanfacine (0.10), Sertraline (0.10), Amphetamine (0.05)
Stimming/perseveration/desire for sameness	Escitalopram (0.04), Oxcarbazepine (0.04), Diazepam (0.03)
Tics/abnormal movements	Guanfacine (0.10), Amphetamine (0.02), Clonazepam (0.02)

The benefit rating is calculated based on the net benefit (overall benefit minus overall adverse) × % of participants reporting that symptom as a primary benefit. So, higher scores suggest more benefit for that symptom. For each symptom, we report the top three rated medications, and continue rating until the score drops <0.1.

OCD, obsessive-compulsive disorder.

This ranking is imperfect because we only ask if the medication benefited the symptoms, not if they had the symptom to begin with. For example, if only a small percentage of the users of a medication had a given symptom such as self-abuse, then even if all of those participants had an improvement the score would still be low, since only a small percentage of the total users benefited. Hence, caution is exercised in interpreting these results, especially for symptoms that are rare in the ASD population, and the tables should only be used as a guide to the most promising medications to consider for a given symptom.

## Discussion

Overall, there was significant variation in net benefit of the medications, with some medications having substantially higher overall benefit scores compared with overall adverse scores, but many medications having only slightly positive or even negative net benefit. It is important to remember that these scores are averages, and individual responses had substantial variation. Table 8 illustrates this for risperidone, showing the percentage of people who rated each symptom level.

**Table 8.** Individual Responses for Risperidone

*Risperidone*			
		N	*%*
Overall benefit rating	No benefit	54	32
	Slight benefit	33	19
	Moderate benefit	35	21
	Good benefit	30	18
	Great benefit	18	11
Overall adverse effect rating	No adverse effects	54	32
	Mild adverse effects	37	22
	Moderate adverse effects	40	24
	Severe adverse effects	39	23

The survey suggests that some medications may have some benefits and/or AEs that have not been evaluated in randomized trials, because those symptoms were not asked about. It is important to remember that most randomized trials are short, and may miss some long-term benefits and AEs. The survey data presumably include some individuals with longer term use, and may offer more insight into longer term benefits and AEs.

It is interesting to note that the most commonly used medications are not necessarily the most highly rated ones; in some cases this may be due to newer, more promising medications not being used much yet.

The results of the study in terms of specific benefits and AEs were often similar to the results of open-label and RDBPCTs, which helps validate this survey. Some of the differences were because the study did not investigate the symptoms that we included in our survey. Table 9 provides a comparison of the symptom improvements reported in the survey and the symptom changes reported in the literature with bold font where there is agreement. In general there is reasonable agreement for many, but not all, symptoms and there are many medications for which there are no data in the literature.

**Table 9.** Comparison of Survey Results with the Literature

*Category*	*Medication*	*Main benefits for individuals with ASD according to the literature data*	*Main benefits according to the survey*
Stimulants	Adderall	N/A	Attention, hyperactivity, cognition
	Amphetamine	N/A	Attention, hyperactivity, anxiety, cognition, irritability
	Dexmethylphenidate/Focalin	N/A	Attention, hyperactivity, cognition
	Methylphenidate/Ritalin/Medate/Concerta	**Hyperactivity**, stereotypy, irritability	Attention, **hyperactivity**, cognition
Antipsychotics	Risperidone/Risperdal	**Aggression**, **irritability**	**Aggression/agitation**, **irritability**, anxiety
	Aripiprazole/Abilify	**Irritability**, **aggression**	**Aggression/agitation**, **irritability**, anxiety
	Olanzapine/Zyprexa	**CGI-I**, irritability, **aggression**, repetitive behavior	**Aggression/agitation**, anxiety, **hyperactivity**
	Quetiapine/Seroquel	**Sleep**, **aggression**, hyperactivity	**Aggression/agitation**, **falling asleep**, **staying asleep**
Antiepileptics	Carbamazepine/Tegretol/Carbatrol Epitol	**Seizures**	**Seizures**, anxiety, irritability
	Clonazepam/Klonopin	Seizures	Anxiety, falling asleep, staying asleep
	Diazepam/Valium	N/A	Anxiety, seizures, aggression/agitation
	Divalproex Sodium/Depakote	Repetitive behaviors, **irritability**, **seizures**, “**behavioral symptoms**”	**Seizures**, **aggression/agitation**, **irritability**
	Gabapentin/Neurontin/Gralise/Horizant	Seizures	Anxiety, general benefit, irritability
	Lamotrigine/Lamictal	**Seizures**	**Seizures**, aggression/agitation, anxiety, irritability
	Levetiracetam/Keppra	**Seizures**, attention, hyperactivity, **aggression**	**Seizures**, **aggression/agitation**, Anxiety, cognition, language/communication, social interaction, and understanding
	Oxcarbazepine/Oxtellar/Trileptal	**Seizures**	Aggression/agitation, **seizures**, anxiety
	Topiramate/Qudexy/Topamax	**irritability**, hyperactivity, stereotypy, anxiety, **depression**	Aggression/agitation, cognition, **depression**, dizziness/unsteadiness, **irritability**
SSRIs	Citalopram/CeleXA	**Irritability**	Anxiety, depression, **irritability**
	Escitalopram/Lexapro	ABC-CV-**irritability**, hyperactivity, stereotypy	Anxiety, depression, general benefit, Aggression/agitation, **irritability**
	Fluoxetine/Prozac/Sarafem/Rapidflux	CGI-I, OCD behaviors, **anxiety**	**Anxiety**, depression, aggression/agitation
	Paxil/Paroxetine	N/A	Anxiety, attention, depression, social interaction, and understanding
	Sertraline/Zoloft	**Aggression**, repetitive behaviors, **anxiety**	**Anxiety**, depression, **aggression/agitation**, irritability
Other	Clonidine/Catapres/Kapvay	Irritability, hyperactivity, stereotypy	Falling asleep, staying asleep, anxiety
	Guanfacine/Intuniv/Tenex	**Hyperactivity**, **attention**	**Attention**, **hyperactivity**, aggression/agitation, cognition
	Buspirone/Buspar/Vanspar	**Irritability**	Anxiety, aggression/agitation, **irritability**
	Atomoxetine/Strattera	**Hyperactivity**, **attention**	**Attention**, anxiety, aggression/agitation, **hyperactivity**

The bold font indicates that both the literature review and the survey data found that benefit.

ABC-CV, aberrant behavior checklist-community version; ASD, autism spectrum disorder; ASDOCD, obsessive-compulsive disorder; CGI-I, clinical global impression of improvement; N/A, no literature data; SSRI, selective serotonin reuptake inhibitor.

Overall, the symptom table shows that medications generally have little effect on core ASD symptoms (language, social interaction, and stereotypic behavior), but do help with some of the comorbid symptoms. In terms of categories of medications, seizure and “other” had several medications with the highest overall benefit scores, followed by antipsychotic and SSRI, with stimulants having the lowest scores.

This study had several advantages over standard clinical trials and previous survey studies:

1. Although less rigorous than a RDBPCT, an online survey is also vastly less expensive, so for less than the cost of one RDBPCT we could collect data on 26 medications from 504 participants.
2. By using the same rating scale for all medications, we were able to compare all 26 medications directly against each other.
3. Compared with previous surveys, which only rated the overall net benefit, this survey provided more details on the overall benefit and overall adverse rating, and provided a listing of the specific symptoms commonly affected.
4. An advantage of this survey is the large number of symptoms evaluated, but a limitation is that the evaluation was limited to a single question.
5. The survey allowed us to collect efficacy data for many medications that have not been studied for people with ASD.
6. The survey included some participants who had been using the medications for a long period of time, so that longer term effects (positive or negative) could be assessed.

There were also some limitations to this type of study:

1. The results are subject to “placebo effect” since it represents clinical data without a placebo control, so the real benefit is likely less than the perceived benefit. Thus, survey data are less reliable than RDBPCTs.
2. The survey is retrospective and based on respondent memory which reduces the accuracy.
3. Dosages were not reported since our early versions suggested that participants did not remember dosages and body weight from years ago. So, the study only represents the doses that were prescribed by the participant's physician.
4. The time of day that the participants took the medications was not reported, and it is possible that the timing of the administration had an effect on efficacy and possible AEs. Further, the survey did not ask if the medications were given in isolation or in a combination that could have affected efficacy.
5. Some medications in the survey have both an immediate release and an extended release formulation, which perform somewhat differently and can have different benefits and AEs.
6. For space reasons, the data are only able to be presented as averages, and as shown in Table 8 there can be a wide range of individual responses to a given medication.
7. The rating scale was slightly asymmetric, with ratings of 0–4 for benefits and 0–3 for AEs, so that the net benefit is slightly biased toward positive benefit. However, since a score of 4 was uncommon, the bias is small.

## Conclusion

Most medications were rated as having a slightly greater benefit than AE. Six medications (lamotrigine, oxcarbazepine, clonidine, guanfacine, buspirone, and sertraline) had overall benefit ratings that were more than twice their overall adverse rating. Conversely, some medications had slightly negative net benefit ratings (worse AEs than benefits on average), including Adderall, Paroxetine, Quetiapine, Olanzapine, and Topiramate. However, there were wide variations in individual ratings of benefit and AEs, suggesting that clinical response to medications was highly variable, so these scores simply represent averages. Autism is heterogeneous, so a medication that helps one person may or may not help another.

A ranking of the top medications (those with the highest net perceived benefit) for each of 18 different symptoms is provided, which may provide some clinical guidance as to which medications might be most worth considering for a given symptom.

A comparison of the survey results with the results of clinical trials shows generally good agreement in terms of medication benefits with some differences; in some cases, the differences are because the clinical trials did not assess all of the symptoms assessed by this survey.

## Clinical Significance

It is hoped that physicians and their patients will find the survey results of use in selecting the most promising medications for a given symptom, and also for monitoring for likely benefits and AEs, especially for medications for which few or no studies have been carried out in ASD populations. It is also hoped that researchers and pharmaceutical companies will find the results useful in designing clinical trials, specifically for selecting appropriate evaluation tools and monitoring for possible AEs. Also, these data suggest that although medications are effective for comorbid symptoms, there is a need for medications that can affect the core symptoms of ASD. Finally, we believe this information may be useful for the FDA in conducting long-term monitoring of safety and efficacy of medications.

## References

[B1] Abdallah MW, Greaves-Lord K, Grove J, Nørgaard-Pedersen B, Hougaard DM, Mortensen EL: Psychiatric comorbidities in autism spectrum disorders: Findings from a Danish Historic Birth Cohort. Eur Child Adolesc Psychiatry 20:599–601, 2011 21971944 10.1007/s00787-011-0220-2

[B2] Adams JB, Holloway CE, George F, Quig D: Analyses of toxic metals and essential minerals in the hair of Arizona children with autism and associated conditions, and their mothers. Biol Trace Elem Res 110:193–210, 2006 16845157 10.1385/BTER:110:3:193

[B3] Adams JB, Romdalvik J, Levine K, Hu L: Mercury in first-cut baby hair of children with autism versus typically-developing children. Toxicol Environ Chem 90:739–753, 2008

[B4] Adams JB, Romdalvik J, Ramanujam VM, Legator MS: Mercury, lead, and zinc in baby teeth of children with autism versus controls. J Toxicol Environ Health A 70:1046–1051, 2007 17497416 10.1080/15287390601172080

[B5] Autism Research Institute: Parent ratings of behavioral effects of biomedical interventions (Publication No. 34). 2009 Available at: https://www.autism.com/pdf/providers/ParentRatings2009.pdf (accessed 823, 2017)

[B6] Bishop JR, Najjar F, Rubin LH, Guter SJ, Owley T, Mosconi MW, Jacob S, Cook EH: Escitalopram pharmacogenetics: CYP2C19 relationships with dosing and clinical outcomes in autism spectrum disorder. Pharmacogenet Genomics 25:548–554, 2015 26313485 10.1097/FPC.0000000000000173 PMC4591203

[B7] Bobo WV, Cooper WO, Stein CM, Olfson M, Graham D, Daugherty J, Fuchs DC, Ray WA: Antipsychotics and the risk of type 2 diabetes mellitus in children and youth. JAMA Psychiatry 70:1067–1075, 2013 23965896 10.1001/jamapsychiatry.2013.2053

[B8] Correll CU, Manu P, Olshanskiy V, Napolitano B, Kane JM, Malhotra AK: Cardiometabolic risk of second-generation antipsychotic medications during first-time use in children and adolescents. JAMA 302:1765–1773, 2009 19861668 10.1001/jama.2009.1549 PMC3055794

[B9] Doyle CA, Mcdougle CJ: Pharmacotherapy to control behavioral symptoms in children with autism. Expert Opin Pharmacother 13:1615–1629, 2012 22550944 10.1517/14656566.2012.674110

[B10] Fombonne E: Epidemiological surveys of autism and other pervasive developmental disorders: An update. J Autism Dev Disord 33:365–382, 2003 12959416 10.1023/a:1025054610557

[B11] Frye RE, Rossignol DA: Identification and treatment of pathophysiological comorbidities of autism spectrum disorder to achieve optimal outcomes. Clin Med Insights Pediatr 10:43–56, 2016 27330338 10.4137/CMPed.S38337 PMC4910649

[B12] Frye RE, Rossignol D, Casanova MF, Brown GL, Martin V, Edelson S, Coben R, Lewine J, Slattery JC, Lau C, Hardy P, Fatemi SH, Folsom TD, Macfabe D, Adams JB: A review of traditional and novel treatments for seizures in autism spectrum disorder: Findings from a systematic review and expert panel. Front Public Health 1:31, 2013 24350200 10.3389/fpubh.2013.00031 PMC3859980

[B13] Frye RE, Sreenivasula S, Adams JB: Traditional and non-traditional treatments for autism spectrum disorder with seizures: An on-line survey. BMC Pediatr 11:37, 2011 21592359 10.1186/1471-2431-11-37 PMC3123184

[B14] Fung LK, Mahajan R, Nozzolillo A, Bernal P, Krasner A, Jo B, Coury D, Whitaker A, Veenstra-Vanderweele J, Hardan AY: Pharmacologic treatment of severe irritability and problem behaviors in autism: A systematic review and meta-analysis. Pediatrics 137(Suppl 2):S124–S135, 2016 10.1542/peds.2015-2851K 26908468

[B15] Ghanizadeh A: Atomoxetine for treating ADHD symptoms in autism. J Atten Disord 17:635–640, 2012 22544388 10.1177/1087054712443154

[B16] Ghanizadeh A, Ayoobzadehshirazi A: A randomized double-blind placebo-controlled clinical trial of adjuvant buspirone for irritability in autism. Pediatr Neurol 52:77–81, 2015 25451017 10.1016/j.pediatrneurol.2014.09.017

[B17] Goin-Kochel RP, Mackintosh VH, Myers BJ: Parental reports on the efficacy of treatments and therapies for their children with autism spectrum disorders. Res Autism Spectr Disord 3:528–537, 2009

[B18] Houghton R, Ong RC, Bolognani F: Psychiatric comorbidities and use of psychotropic medications in people with autism spectrum disorder in the United States. Autism Res 10:2037–2047, 2017 28963809 10.1002/aur.1848

[B19] Jaselskis CA, Cook EH, Fletcher KE, Leventhal BL: Clonidine treatment of hyperactive and impulsive children with autistic disorder. J Clin Psychopharmacol 12:322–327, 1992 1479049

[B20] King BH, Hollander E, Sikich L, McCracken JT, Scahill L, Bergman JD, Donnelly CL, Anagnostou E, Dukes K, Sullivan L, Hirtz D, Wagner A, Ritz L: Lack of efficacy of citalopram in children with autism spectrum disorders and high levels of repetitive behavior. Arch Gen Psychiatry 66:583–590, 2009 19487623 10.1001/archgenpsychiatry.2009.30 PMC4112556

[B21] Kolevzon A, Mathewson KA, Hollander E: Selective serotonin reuptake inhibitors in autism. J Clin Psychiatry 67:407–414, 2006 16649827 10.4088/jcp.v67n0311

[B22] Konstantareas MM, Homatidis S: Brief report: Ear infections in autistic and normal children. J Autism Dev Disord 17:585–594, 1987 3680158 10.1007/BF01486973

[B23] LeClerc S, Easley D: Pharmacological therapies for autism spectrum disorder: A review. P T 40:389–397, 2015 26045648 PMC4450669

[B24] Masi G, Milone A, Veltri S, Iuliano R, Pfanner C, Pisano S: Use of quetiapine in children and adolescents. Pediatr Drugs 17:125–140, 2015 10.1007/s40272-015-0119-3 25686575

[B25] Ming X, Gordon E, Kang N, Wagner GC: Use of clonidine in children with autism spectrum disorders. Brain Dev 30:454–460, 2008 18280681 10.1016/j.braindev.2007.12.007

[B26] Niehus R, Lord C: Early medical history of children with autism spectrum disorders. J Dev Behav Pediatr 27:S120–S127, 2006 16685178 10.1097/00004703-200604002-00010

[B27] Owen-Smith AA, Bent S, Lynch FL, Coleman KJ, Yau VM, Pearson KA, Massolo ML, Quinn V, Creon LA: Prevalence and predictors of complementary and alternative medicine use in a large insured sample of children with autism spectrum disorders. Res Autism Spectr Disord 17:40–51, 2015 26366192 10.1016/j.rasd.2015.05.002 PMC4562462

[B28] Reichow B, Volkmar FR, Bloch MH: Systematic review and meta-analysis of pharmacological treatment of the symptoms of attention-deficit/hyperactivity disorder in children with pervasive developmental disorders. J Autism Dev Disord 43:2435–2441, 2013 23468071 10.1007/s10803-013-1793-z PMC3787525

[B29] Research Units on Pediatric Psychopharmacology Autism Network: Randomized, controlled, crossover trial of methylphenidate in pervasive developmental disorders with hyperactivity. Arch Gen Psychiatry 62:1266–1274, 2005 16275814 10.1001/archpsyc.62.11.1266

[B30] Sochocky N, Milin R: Second generation antipsychotics in Asperger's disorder and high functioning autism: A systematic review of the literature and effectiveness of meta-analysis. Curr Clin Pharmacol 8:370–379, 2013 24050741 10.2174/15748847113086660073

[B31] Williams K, Brignell A, Randall M, Silove N, Hazell P: Selective serotonin reuptake inhibitors (SSRIs) for autism spectrum disorders (ASD). Cochrane Database Syst Rev (8):CD004677, 2013 23959778 10.1002/14651858.CD004677.pub3 PMC11990048

[B32] Wink LK, Erickson CA, Mcdougle CJ: Pharmacologic treatment of behavioral symptoms associated with autism and other pervasive developmental disorders. Curr Treat Options Neurol 12:529–538, 2010 20848330 10.1007/s11940-010-0091-8

